# microRNAs Tune Oxidative Stress in Cancer Therapeutic Tolerance and Resistance

**DOI:** 10.3390/ijms20236094

**Published:** 2019-12-03

**Authors:** Wen Cai Zhang

**Affiliations:** Burnett School of Biomedical Sciences, College of Medicine, University of Central Florida, 6900 Lake Nona Blvd, Orlando, FL 32827, USA; wencai.zhang@ucf.edu; Tel.: +1-(407) 266-7178

**Keywords:** microRNA, cancer, oxidative stress, reactive oxygen species, redox signaling, hypoxia, therapeutic tolerance, therapeutic resistance

## Abstract

Relapsed disease following first-line therapy remains one of the central problems in cancer management, including chemotherapy, radiotherapy, growth factor receptor-based targeted therapy, and immune checkpoint-based immunotherapy. Cancer cells develop therapeutic resistance through both intrinsic and extrinsic mechanisms including cellular heterogeneity, drug tolerance, bypassing alternative signaling pathways, as well as the acquisition of new genetic mutations. Reactive oxygen species (ROSs) are byproducts originated from cellular oxidative metabolism. Recent discoveries have shown that a disabled antioxidant program leads to therapeutic resistance in several types of cancers. ROSs are finely tuned by dysregulated microRNAs, and vice versa. However, mechanisms of a crosstalk between ROSs and microRNAs in regulating therapeutic resistance are not clear. Here, we summarize how the microRNA–ROS network modulates cancer therapeutic tolerance and resistance and direct new vulnerable targets against drug tolerance and resistance for future applications.

## 1. Reactive Oxygen Species (ROSs)

There are many types of free radicals including oxygen- and nitrogen-based species. ROSs or reactive oxygen metabolites are free radicals containing oxygen metabolites such as single oxygen, the superoxide anion, hydrogen peroxide, and the hydroxyl radical [1]. ROSs are generated from cellular oxidative metabolism, including mitochondrial oxidative phosphorylation and electron transfer reactions, and optimal levels of ROSs play a pivotal role in many cellular functions [2]. At physiological levels, ROSs are considered signaling molecules or secondary messengers that participate in cell signal transduction, a process known as redox signaling [3]. In addition, the production of ROSs by phagocytic cells is recognized as an important part of innate immunity that kills invading pathogens [4].

The coordination between ROS generation and scavenging ensures that ROS levels are tightly controlled and fine-tuned so as to act as secondary messengers for cell signaling [5]. However, the aberrant production of ROSs, or the failure of the capacity to scavenge excessive ROSs, results in an imbalance in the redox environment of the cell [6]. High levels of ROSs have deleterious effects including nucleic acid (DNA and RNA), lipid, and protein oxidation, as well as membrane destruction by lipid peroxide formation, leading to the development of various diseases such as cancer [7]. Using antioxidant-based strategies [8] to decrease ROS levels or inhibit oxidative damage may prevent ROS-induced cell damage. For example, peroxisome proliferator-activated receptor-gamma coactivator 1 alpha upregulates expression levels of superoxide dismutase enzymes (SOD2/SOD3) and catalase to protect cells from oxidative damage via detoxification and DNA repair [9].

Aberrantly regulated metabolic pathways lead to tumorigenesis [10] and preferential survival of tumor cells [11]. Accumulating evidence suggests that tumorigenesis is dependent on mitochondrial metabolism [12], especially the tricarboxylic acid (TCA) cycle [13]. The TCA cycle is a central pathway in the metabolism of sugars, lipids, and amino acids [14]. Dysregulation of the TCA cycle can induce oncogenesis by activating pseudohypoxia responses, which result in the expression of hypoxia-associated proteins irrespective of oxygen status [15]. For example, succinate accumulation caused by functional loss of the TCA cycle enzyme succinate dehydrogenase complex stabilizes hypoxia-inducible factor (HIF)-1α via inhibition of prolyl hydroxylase (PHD) [16]. In addition, loss of function of the von Hippel–Lindau (VHL) protein [17] also induces pseudohypoxia responses through decreased ubiquitination and proteasomal degradation of HIF-1α [18]. Among the 1158 mitochondrial genes discovered in MitoCarta2.0 (Broad Institute) [19,20], the succinate dehydrogenase complex [21] inclusive of succinate dehydrogenase A [22], succinate dehydrogenase B [23], succinate dehydrogenase C [24], and succinate dehydrogenase D [25], as well as glycine decarboxylase [26,27,28,29] and glutaminase [30], is especially critical for tumorigenesis. Hypoxia, acting through HIF-1α, results in a low production of ROSs and high antioxidant defense in cancers such as leukemia [31]. It suggests that targeting key enzymes of hypoxia metabolism pathways might provide a new way to eradicate tumor formation [32]. 

## 2. microRNAs (miRNAs)

miRNAs are important regulators of mRNA expression [33] and play critical roles in regulating tumor initiation and progression [34]. Importantly, single miRNAs have been shown to regulate entire cell signaling networks in a cell-context dependent manner [35] and may also be utilized as biomarkers [36,37,38] for both invasive [39,40] and non-invasive [41,42,43] detection. Dysregulated expressions of miRNAs may function as oncogenes (oncomiRs) [44] such as miR-21 [45], miR-31 [46], miR-155 [47,48], and miR-10b [49] or as tumor suppressors such as *let-7* [50] and miR-34 [51,52] in many cancers. 

ROSs are finely tuned by dysregulated miRNAs, and vice versa. Many studies are focused on regulatory interactions between miRNAs and ROSs attributing to oxidative stress-related tissue [53]. It is important for a well-regulated cellular ROS level, and miRNAs fill in the role of maintaining this homeostasis. A dysregulation of normal physiological miRNA levels can thus lead to oxidative damage and the development of diseases such as cancer. For example, oncogenic miR-21 enhances both KRAS [54] and epidermal growth factor receptor (EGFR) signaling [55] and promotes tumorigenesis through stimulation of mitogen-activated protein kinase (MAPK)-mediated ROS production by downregulation of SOD2/SOD3 [56]. On the other hand, oxidative stress can alter the expression level of many miRNAs [57,58,59]. For instance, oxidative stress such as hydrogen peroxide elevates miR-34a with concomitant reduction of sirtuin-1 and sirtuin-6 in bronchial epithelial cells [60], which is associated with chronic obstructive pulmonary disease and tumorigenesis [61]. However, oxidative stress decreases expression levels of the *let-7* family [62] in a p53-dependent manner in a variety of tumor cells [63]. These findings suggest that ROSs may exert a pivotal role in the regulation of microRNA expression in a cell-context-dependent manner.

miRNA-based monotherapy has not been developed well in clinical settings [64,65,66]. For example, a first-in-man, phase 1 clinical trial of miR-16-loaded nanoparticles as a treatment for recurrent malignant pleural mesothelioma patients has been completed [67]. Delivery of tumor suppressive miR-16 in 22 patients led to 5% objective response, 68% stable disease, and 27% progressive disease. Possible mechanisms of low objective response include miRNA sequestration through leaky cancer blood vessels as well as endocytosis by cancer cells [68]. Nevertheless, miR-16 expression levels in patients should be detected prior to receiving miR-16 treatment in future clinical trials [69]. Furthermore, miRNA-based treatment may combine with other current or potential therapeutics in combating cancer [70,71]. In addition, increasing evidence has revealed that miRNAs can be directly linked to therapeutic resistance in some cancers. For instance, overexpressing miR-205 sensitizes radioresistant breast cancer cells to radiation in a xenograft model [72]. Similarly, administration of miR-24 sensitizes radioresistant nasopharyngeal carcinoma cells to radiation in vitro [73]. miRNA-mediated regulation of signaling pathways involved in tumorigenesis as well as therapeutic tolerance and resistance is summarized in Table 1. It is revealed that miRNAs may serve both as drug targets and as therapeutic agents to eradicate cancer cells and sensitize therapeutic resistant cells [74].

## 3. Therapeutic Tolerance and Resistance

The discovery of genetic mutation on tyrosine kinase, such as *EGFR* mutations including exon 19 deletion (*Del19*) and exon 21 Leu858Arg substitution (*L858R*), that confer sensitivity to EGFR-targeted tyrosine kinase inhibitors in lung adenocarcinomas heralded the beginning of the era of precision medicine for lung cancer [91,92]. However, the success of EGFR-based therapy was compromised by therapeutic resistance following initial treatment response in most cancer patients [93]. Exon 20 Thr790Met substitution (*T790M*), affecting the ATP binding pocket of the EGFR kinase domain, accounts for approximately half of all lung cancer cases with acquired resistance to the current first generation EGFR tyrosine kinase inhibitors, erlotinib and gefitinib [94]. In erlotinib- and gefitinib-resistant lung tumors with *EGFR^T790M^*, rociletinib and osimertinib are highly active [95]. However, resistance to the third generation EGFR tyrosine kinase inhibitor osimertinib is now emerging clinically [96]. In addition to genetic mutations, intratumor heterogeneity also drives neoplastic progression and therapeutic resistance [97]. Recently, it has been found that *EGFR^T790M^*-positive drug-resistant cells are derived from *EGFR^T790M^*-negative drug-tolerant persister cells that survive initial EGFR tyrosine kinase inhibitors treatment [98,99]. It is therefore crucial to identify molecular changes that drive drug tolerance.

Consistently, Zhang et al. have revealed that lung tumor cells protect themselves with a drug-tolerance mechanism when the cells are treated with osimertinib [76]. These findings align with previous data showing that tumor cells enter into a tolerant state when they are treated with tyrosine kinase inhibitors in lung and other cancers [100,101,102]. These tolerant persister cells precede and evolve into resistant cells over time by acquiring *EGFR*-resistant mutations [98,99]. These tolerant cells are slow cycling and are enriched in the expression of stem-associated genes in the WNT/planar cell polarity signaling pathway, such as *WNT5A*, *FZD2*, and *FZD7*. These findings are conceptually similar to a recent report that post-drug transition to stable resistance consists of dedifferentiation [103]. 

Excessive ROSs produced by damaged mitochondria can trigger mitophagy, a process that can scavenge impaired mitochondria and reduce ROS levels to maintain a stable mitochondrial function in cells [104]. Therefore, mitophagy helps maintain cellular homeostasis under oxidative stress. For example, protein kinase inhibitor sorafenib shows activities against many protein kinases, including vascular endothelial growth factor receptor (VEGFR), platelet-derived growth factor receptor (PDGFR), and rapidly accelerated fibrosarcoma (RAF) kinases [105]. Resistance to sorafenib in cancers such as hepatocellular carcinoma is frequent [106] partially due to antiangiogenic effects-mediated hypoxia [107]. Administration of tryptophan-derived metabolites such as melatonin [108] increased ROS production and mitophagy, resulting in increased sensitivity to sorafenib in hepatocellular carcinoma cells [109]. Additionally, melatonin downregulated the HIF-1α protein synthesis through inhibition of the mammalian target of rapamycin complex 1 (mTORC1)-mediated pathway [110]. Most recently, it was shown that drug-tolerant persister cancer cells were vulnerable to inhibition of the glutathione peroxidase 4, owing to a disabled antioxidant program [102]. It suggests that increasing ROS levels may re-sensitize therapeutic resistant cancer cells to current treatments. 

## 4. miRNA–ROS Interaction Regulates Therapeutic Tolerance/Resistance at the Phenotypic Level

**Figure 1:**
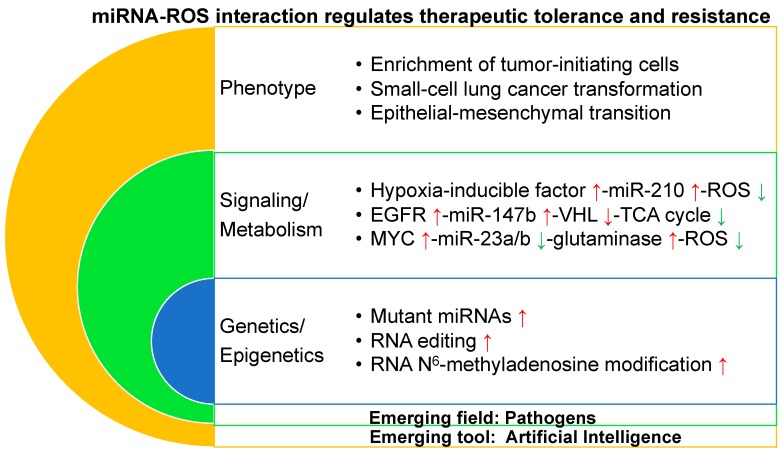
miRNA–ROS interaction regulates cancer therapeutic tolerance and resistance through heterogeneous mechanisms. The mechanisms at hierarchy levels include phenotypic, signaling/metabolic, and genetic/epigenetic changes. ROS: reactive oxygen species; HIF: hypoxia-inducible factor; EGFR: epidermal growth factor receptor; VHL: von Hippel–Lindau; TCA: tricarboxylic acid; ↑: upregulation; ↓: downregulation.

The miRNA–ROS network in a scenario of therapeutic tolerance/resistance is grouped at three levels including phenotype, signaling/metabolism, and genetics/epigenetics (Figure 1). Phenotypic changes include the enrichment of tumor-initiating cells, the histological transformation from *EGFR*-mutant non-small cell lung cancer to small cell lung cancer, and epithelial–mesenchymal transition resulting in therapeutic tolerance/resistance.

### 4.1. Enrichment of Tumor-Initiating Cells

Therapeutic resistance is frequent after primary and adjuvant cancer therapy, often evolving into a lethal relapse disease [111]. These observations may be attributed to the highly heterogeneous nature of tumors that contain distinct tumoral and microenvironment cells, all of which contribute in varying degrees toward self-renewal, drug resistance, and relapse [112]. The tumor-initiating cell or cancer stem cell model provides one explanation for the phenotypic and functional diversity among cancer cells in some tumors [113]. Tumor-initiating cells have been demonstrated to be more resistant to conventional therapeutic interventions [114] and are key drivers of relapse in many types of cancers including leukemia [115], lung cancer [116], breast cancer [117], brain cancer [118], colon cancer [119], and nasopharyngeal carcinoma [120]. There is, therefore, increasing interest in developing strategies that can specifically target tumor-initiating cells with novel and emerging therapeutic modalities, thereby halting cancer progression and improving disease outcome [121]. Tumor-initiating cells protect their genomes from ROS-mediated damage [122] via increased production of free radical scavengers [123] leading to low ROS levels [124]. Thus, heterogeneity of ROS levels in cancers such as glioma may influence the extent to which tumor-initiating cell-enriched populations are resistant to therapies such as ionizing radiation [125]. Tumor-initiating cells display heterogeneous phenotypes due to different genotypes in tumors [126]. Thus, the genetic backgrounds, such as mutant *EGFR* and *RAS*, need to be taken into consideration to better understand the association between tumor-initiating cells and therapeutic resistance in the future.

In non-small cell lung cancer, a panel of tumor-initiating cell-relevant miRNAs is enriched when assessed by a miRNA microarray [75]. Those top upregulated miRNAs include miR-1290 and miR-1246 (Table 1). The top downregulated miRNAs comprise miR-23a and *let-7b/c/d/i*. Further analysis showed that miR-1246 and miR-1290 regulate tumor-initiating cells via repressing cysteine-rich metal-binding proteins (metallothioneins) [75]. The reduced expression of metallothioneins has been implicated as biomarkers of low ROSs, which is consistent with the previous finding that pharmacological anti-oxidants such as N-acetyl cysteine or the knock-down of *nuclear respiratory factor 2* (*NRF2*) prevented the induction of metallothionein-1 induced by tyrosine kinase inhibitor sorafenib [127]. Another direct target of miR-1290, glioma pathogenesis-related protein 1, promotes apoptosis through upregulating ROS production by activating the c-Jun-NH(2) kinase signaling cascade in cancer cells [128]. Other evidence has shown that extracellular miR-1246 could enhance radioresistance of lung cancer cells [129]. In addition, miR-21 is enriched in tumor-initiating cells in many types of cancers such as gastric and breast cancers [130]. Functional loss of miR-21 reduces a frequency of tumor-initiating cells, consistently with decreased capacity of therapeutic resistance against EGFR tyrosine kinase inhibitors [82] (Table 1). Whether these miRNAs regulate ROSs resulting in therapeutic tolerance and resistance still needs further study. Thus, targeting enriched tumor-initiating cells might overcome miRNA–ROS-mediated therapeutic tolerance/resistance. 

### 4.2. Small Cell Lung Cancer Transformation

Small cell lung cancer is a highly aggressive disease that exhibits rapid growth and genetic instability including inactivated *tumor suppressor retinoblastoma 1* (*RB1*) and amplified *MYC proto-oncogene* (*MYC*) [131]. Histologic transformation of *EGFR* mutant non-small cell lung cancer to small cell lung cancer is an important mechanism of resistance to EGFR tyrosine kinase inhibitors that occurs in approximately 3–10% of *EGFR* mutant non-small cell lung cancers [132]. Transformation to small cell lung cancer occurs in a subpopulation of *EGFR* mutant non-small cell lung cancer patients and is frequently associated with mutant *RB1*, *TP53*, and *PIK3CA* [133,134]. Future studies might help define which subsets of non-small cell lung cancer are most prone to small cell lung cancer transformation.

Frequent overexpression of the miR-17~92 cluster in small cell lung cancer [135] is a fine-tuner to reduce excessive ROS-induced DNA damage in RB1-inactivated small cell lung cancer cells [136]. Therefore, miR-17~92 may be excellent therapeutic target candidates to overcome small cell lung cancer transformation.

### 4.3. Epithelial–Mesenchymal Transition

An epithelial–mesenchymal transition is a biologic process that allows a polarized epithelial cell to undergo multiple biochemical changes that enable it to assume a mesenchymal cell phenotype, which includes increased resistance to apoptosis [137]. Epithelial–mesenchymal transition is tightly regulated by microRNAs. For example, downregulation of miR-200 family members is linked to enhanced epithelial–mesenchymal transition and tumor-initiating cell acquisition [138,139] in many cancers [140]. Reduced miR-200s directly increase p38α [141], leading to decreased levels of ROSs and subsequent inactivation of the NRF2 oxidative stress response pathway [142]. The decreased ROSs, in turn, inhibit expression of the miR-200s [143], thus establishing a miR-200s-activated stress signature, which strongly correlates with shorter patient survival caused by chemotherapeutic resistance. In addition, miR-30b/c and miR-222 mediate gefitinib-induced apoptosis and the epithelial–mesenchymal transition leading to therapeutic resistance in non-small cell lung cancer [87]. These discoveries collectively indicate potential roles of the miRNA family in the regulation of ROS homeostasis in tumor-initiating cells and therapeutic resistance.

## 5. miRNA–ROS Interaction Regulates Therapeutic Tolerance/Resistance at a Signaling/Metabolic Level

### 5.1. HIF-miR-210-ROS

Under hypoxic conditions, upregulated HIF-1α directly binds to a hypoxia-responsive element on the proximal miR-210 promoter and induces miR-210 expression in cancer cells [144]. miR-210 activates generation of ROSs [145] via suppressing iron–sulfur cluster assembly enzyme [146,147] and cytochrome c oxidase assembly protein [148] in the mitochondria electron transport chain and the TCA cycle. miR-210 knockdown decreased resistance to radiotherapy in hypoxic glioma stem cells and hepatoma cells [149,150]. These discoveries suggest that the HIF-miR-210-ROS [151] pathway might be a target to overcome therapeutic resistance (Figure 1).

### 5.2. EGFR-miR-147b-VHL-TCA Cycle

Increasing evidence suggests that the metabolic enzymes and the catalyzed metabolites, such as isocitrate dehydrogenase, succinate dehydrogenase, and succinate [16,152] in the TCA cycle, are involved in not only tumorigenesis but also therapeutic resistance. A hypoxia response is linked to tumor cell survival and drug-resistance in many cancers [153,154]. Dysregulated cancer metabolism has recently gained attention for its potential role in promoting therapeutic resistance by a therapeutic tolerance strategy in a novel manner [102]. Furthermore, Zhang et al. discovered that lung cancer cells adopt a tolerance strategy to protect from EGFR tyrosine kinase inhibitors by modulating miR-147b-dependent pseudohypoxia signaling pathways [76]. The study revealed that VHL [155] and succinate dehydrogenase play roles in tolerance-mediated cancer progression. Decreasing miR-147b and reactivation of the TCA cycle pathway provides a promising strategy to prevent therapeutic tolerance-mediated tumor relapse (Figure 1).

In addition, VHL regulates Akt activity [156], suggesting that miR-147b-VHL axis might confer therapeutic tolerance through activating Akt activity. In addition, other upstream transcription factors such as the inhibitor of DNA binding 2 might regulate VHL levels [157]. The interaction between miR-147b and other transcription factors controlling VHL needs to be investigated in the future.

Furthermore, the reciprocal changes of metabolites in the TCA cycle such as increased levels of succinate and 2-oxoglutarate (also known as α-ketoglutarate) [158] as well as decreased levels of malate and fumarate in osimertinib-tolerant cells indicate that silenced activity for succinate dehydrogenase is linked to therapeutic tolerance. In addition, small molecule inhibitor R59949 silencing succinate dehydrogenase activity enhances therapeutic tolerance, which is comparable to the function of miR-147b overexpression in tolerant persister cells. It is not surprising that accumulated succinate due to a loss of function of succinate dehydrogenase could activate the pseudohypoxia signaling pathway by repressing PHD2 as reported previously [16]. This is consistent with the findings that the miR-147b/succinate dehydrogenase axis could increase the gene expression for pseudohypoxia signaling pathways. In addition to inactivated VHL and succinate dehydrogenase, other factors such as reduced nicotinamide adenine dinucleotide (NAD^+^) and decreased glutathione [159] might also activate pseudohypoxia responses leading to therapeutic tolerance. In addition, these pseudohypoxia responses may further perturb the TCA cycle and cooperatively regulate therapeutic tolerance. 

These discoveries suggests that miR-147b may promote drug-tolerance to EGFR tyrosine kinase inhibitors either through reactivation of the EGFR downstream signaling pathway or through bypass by another receptor tyrosine kinase that sustains downstream signaling despite inhibition of EGFR [160,161]. 

### 5.3. Myc-miR-23a/b-Glutaminase-ROS

Cancer cells depend on both glycolysis and glucose oxidation to support their growth [162,163] as well as glutaminolysis that catabolizes glutamine to generate ATP and lactate [164]. Oncogenic c-Myc represses miR-23a and miR-23b, resulting in increased levels of mitochondrial glutaminase in cancer cells [30]. Glutaminase converts glutamine to glutamate, which is further catabolized through the TCA cycle for the production of adenosine triphosphate (ATP) or serves as substrate for glutathione synthesis [165]. Glutamine withdrawal or glutaminase knockdown resulted in increased levels of ROSs. Thus, the Myc-miR-23-glutaminase axis provides a new mechanism for regulating ROS homeostasis in cancer cells. Considering that downregulated miR-23a is enriched in tumor-initiating cells [75], it is of great interest to explore a link between miR-23 and ROSs in therapeutic tolerance/resistance (Figure 1).

## 6. miRNA–ROS Interaction Regulates Therapeutic Tolerance/Resistance at a Genetic/Epigenetic Level

### 6.1. Mutant miRNAs

The whole genome sequencing analysis of lung adenocarcinomas showed noncoding somatic mutational hotspots near *vacuolar membrane protein 1/MIR21* [166]. Samples harboring indels or single nucleotide variants in this locus demonstrated significantly higher levels of *MIR21* expression. miR-21 high levels are linked to therapeutic resistance to several treatments, including EGFR tyrosine kinase inhibitors [167] and chemotherapeutic agents [168]. Thus, it is valuable to predict therapeutic response by detecting the sequence of miR-21 in biopsies from cancer patients before they receive treatments such as EGFR tyrosine kinase inhibitors (Figure 1).

### 6.2. RNA Editing

Adenosine deaminases acting on RNA (ADARs) convert adenosine to inosine in double-stranded RNA including both protein-coding [169] non-coding RNAs [170]. ADAR editase activation has been associated with progression of a broad array of malignancies including therapeutic resistance [171]. ADAR1 promotes tumor-initiating cell activity [172] and resistance to BCR-ABL1 inhibitor or janus kinase 2 inhibitor in chronic myeloid leukemia through inactivating biogenesis of the *let-7* [173] or pri-miR-26a maturation [174]. In addition, most cancer patients either do not respond to the immune checkpoint blockade or develop resistance to it, often because of acquired mutations [175] that impair antigen presentation [176]. Loss of function of ADAR1 in tumor cells profoundly sensitizes tumors to immunotherapy and overcomes resistance to the programmed cell death protein 1 (PD-1) checkpoint blockade [177]. It is of interest to further study how the ADAR-miRNA axis regulates therapeutic tolerance/resistance through controlling potential genes encoding ROS scavengers [178] such as *Drosophila homolog of the mammalian protein thioredoxin-1* and *cytochrome P450 4g1* (Figure 1).

### 6.3. RNA m^6^A Modification

N^6^-methyladenosine (m^6^A) modification of mRNA (RNA m^6^A modification) is the most abundant RNA modification in eukaryotes and highly conserved among multiple species [179]. RNA m^6^A modification is emerging as an important regulator of gene expression that affects different developmental and biological processes [180], and altered m^6^A homeostasis is linked to cancer [181,182,183]. RNA m^6^A modification is catalyzed by the dynamic regulation of methyltransferases and demethylases. Methyltransferase include methyltransferase-like 3 (METTL3), METTL14, and Wilms’ tumor 1-associating protein, and the demethylases include fat mass- and obesity-associated protein and ALKB homolog 5 [184]. Upregulation of METTL3 is associated with poor prognosis in tumorigenesis and increased chemo- and radio-resistance in cancers such as glioblastomas [185] and pancreatic cancer [186]. Developing resistant phenotypes during tyrosine kinase inhibitor therapy is controlled by m^6^A modification [187]. Leukemia cells with mRNA m^6^A demethylation are more tolerant to tyrosine kinase inhibitor treatment. Recovery of m^6^A methylation re-sensitizes therapeutic resistant cells towards tyrosine kinase inhibitors. The findings identify a novel function for the m^6^A methylation in regulating reversible tyrosine kinase inhibitor-tolerance state, providing a mechanistic paradigm for drug resistance in cancer. In addition, METTL3 plays roles in the maturation process of miRNAs against ROSs in an m^6^A-dependent manner [188]. For example, METTL3-mediated miR-873 upregulation controls the kelch-like ECH associated protein 1 (KEAP1)-NRF2 [142] pathway against ROSs. These studies revealed that RNA m^6^A might regulate therapeutic tolerance/resistance through miRNA–ROS pathways (Figure 1).

## 7. Emerging Fields and Tools in Preventing and Overcoming Therapeutic Tolerance/Resistance

### 7.1. Artificial Intelligence (AI)

AI is an area of computer science that emphasizes the creation of intelligent machines that work and react like humans and that uses labeled big data along with markedly enhanced computing power and cloud storage [189]. The most common applications of AI in drug treatment have to do with matching patients to their optimal drug or combination of drugs, predicting drug–target or drug–drug interactions and optimizing treatment protocols [190]. AI-based models have been developed for predicting synergistic treatment combinations in many diseases such as infectious diseases [191] and cancers [192,193]. One challenge is determining how AI-based technology may design tools which improve identification of therapeutic tolerance and resistance and develop new treatment combinations against tolerant and resistant cancers. The success of this AI-based approach may provide earlier and targeted anticancer treatment, which would prevent therapeutic tolerance/resistance emerging and cure cancer patients more effectively (Figure 1).

### 7.2. Pathogens

Pathogens such as microbiomes and viruses are becoming increasingly recognized for their effects on tumorigenesis and therapeutic resistance to cancer treatment [194]. Bacterial dysbiosis accompanies carcinogenesis in several malignancies such as gastric [195], colon [196], liver [197], and pancreatic [198] cancers by affecting metabolism and impairing immune functions [199]. Additionally, fungi [200] and viruses [201] also induce carcinogenesis in several cancers. Furthermore, intratumoral bacteria induced therapeutic resistance through breakdown of chemotherapy gemcitabine into inactive metabolites via bacterial enzymes such as cytidine deaminase [202] and via impairing response to immune checkpoint blockade [198]. Gut microbiota plays a critical role in mediating colorectal cancer chemoresistance in response to chemotherapeutics via a selective target loss of miR-18a* (miR-18a-3p) and miR-4802, and via activation of the autophagy pathway [203]. In addition, miR-18a* is a tumor suppressor that inhibits KRAS expression [204]. Activating *KRAS* mutations confer both primary [205] and acquired [206] resistance to anti-EGFR cetuximab therapy in colorectal cancer. Thus, targeting intratumoral pathogens provide a new angle in cancer treatment to overcome therapeutic tolerance/resistance. Some intracellular pathogens interact directly with receptor tyrosine kinases, and this interaction is critical for pathogen entry [207]. This establishes that pathogen-encoded receptor tyrosine kinase-interacting epitopes represent promising candidates for the development of novel therapeutic and prophylactic vaccines and of small-molecule interaction disruptors [208]. It would be of great interest to investigate whether those pathogens will confer therapeutic tolerance/resistance in host tumor cells by regulating miRNA–ROS interaction (Figure 1).

## 8. Concluding Remarks and Future Directions

Therapeutic tolerance/resistance raise major problems for the successful treatment of cancer, including conventional therapy and recent molecular therapy. There is an increasing importance of studying the role of ROS-relevant miRNAs to identify more effective biomarkers and develop better therapeutic targets against therapeutic tolerance/resistance. The interaction between miRNAs and ROSs fits in with the opportunities and challenges of studying mechanisms by which cancer cells resist therapy and ways by which therapeutic tolerance/resistance can be overcome. New concepts and emerging research tools bring potential to overcome therapeutic tolerance/resistance. However, some major challenges should be addressed properly. First, cancer relapse is driven by a small subpopulation of drug-tolerant persister cells, known as minimal residual disease in clinic. Single cell-relevant technologies, such as single-cell sequencing [209] might be applied to track single tolerant persister cells to gain insights into drug tolerance dynamics and heterogeneity [210]. In addition, preventative strategies using potential agents targeting those therapeutic tolerant cells at early stages in combination with molecular therapeutics will help prevent therapeutic tolerance and the resulting therapeutic resistance [211]. Second, new ex vivo models such as the organoid have been widely applied in cancer treatment response and therapeutic tolerance/resistance [212,213]. One of the advantages of the three-dimensional organoid model compared to a conventional two-dimensional monolayer is that tumor microenvironments established in organoids are similar to those found in vivo. For example, cancer organoids show heterogeneous hypoxic regions and show their enriched tumor-initiating cells and relevant metabolism pathway [214]. The organoid model may be used for large-scale screening, especially when incorporated with AI-based technology, to optimize the best drug combinations and thus reduce therapeutic tolerance/resistance. However, lacking immune cells and other types of cells has challenged this model [215]. Thus, incorporating immune cells will help better understand tolerance and resistance to immunotherapy [216]. Third, applications of non-invasive biomarkers to predict drug response represents a future direction in clinical settings. For example, cell-free circulating miRNAs have been successfully combined with low dose computed tomography scanning for diagnoses of early-stage lung cancer patients [217]. It is reasonable to incorporate cell-free circulating miRNAs signature together with cell-free DNAs signature [218] to predict and track the emergence of therapeutic tolerance/resistance. However, microRNAs predicting therapeutic tolerance/resistance might be dependent on specific mutant driver genes. For instance, increased miR-147b is relevant to mutant *EGFR* [76], and downregulated miR-23a is relevant to mutant *MYC* [30]. Thus, genetic mutation background and specific treatment agents should be considered comprehensively. Ultimately, early intervention on genetic/epigenetic, signaling/metabolic, and phenotypic changes in the miRNA–ROS network should be considered comprehensively to prevent and overcome therapeutic tolerance/resistance.

## Figures and Tables

**Table 1:** miRNA-mediated regulation of signaling pathways involved in tumorigenesis as well as therapeutic tolerance and resistance.

miRNA	Signaling Involved in Tumorigenesis	Signaling Involved in Therapeutic Tolerance and Resistance
miR-1246 and miR-1290 ↑	(+) tumorigenesis via repressing metallothioneins in human non-small cell lung cancer [75]	(+) resistance to EGFR tyrosine kinase inhibitor gefitinib via repressing metallothioneins in human non-small cell lung cancer [75]
miR-147b ↑	N.A.	(+) tolerance to EGFR tyrosine kinase inhibitor osimertinib through activating pseudohypoxia signaling pathways via repressing VHL and succinate dehydrogenase in human non-small cell lung cancer [76]
miR-155 ↑	(+) tumorigenesis in mouse miR155 transgenic B cell lymphomas [77]	(+) chemoresistance to gemcitabine through decreasing apoptosis in human pancreatic cancer [78]
miR-21 ↑	(+) Ras/MEK/ERK signaling via repressing negative regulators of the Ras/MEK/ERK pathway and inhibition of apoptosis in mouse KRAS transgenic non-small cell lung cancer [54]	(+) chemoresistance to gemcitabine through decreasing apoptosis and activating Akt phosphorylation in human pancreatic cancer [79,80] (+) radioresistance through upregulation of hypoxia-inducible factor 1α in human non-small cell lung cancer [81] (+) resistance to EGFR tyrosine kinase inhibitors through activating PI3K-AKT signaling pathway in human non-small cell lung cancer [82]
miR-31 ↑	(+) tumorigenesis through activating RAS/MAPK signaling via repressing negative regulators of RAS/MAPK signaling in mouse *KRAS* transgenic non-small cell lung cancer [46]	N.A.
let-7 family ↓	(+) tumorigenesis in human breast cancer through repressing H-RAS and high mobility group AT-hook 2 [83]	(+) resistance to EGFR tyrosine kinase inhibitor gefitinib through upregulation of MYC in human non-small cell lung cancer [84]
miR-30 ↓	(+) tumor initiation and (−) apoptosis by repressing ubiquitin-conjugating enzyme 9 and integrin beta3, respectively, in human breast cancer [85] (+) mTOR/AKT-signaling pathway through repressing transmembrane 4 super family member 1 in human non-small cell lung cancer [86]	(−) resistance to EGFR tyrosine kinase inhibitor gefitinib through repressing BCL2-like 11 and apoptotic peptidase activating factor 1 in human non-small cell lung cancer [87] (+) chemoresistance to cisplatin through activating autophagy in human gastric cancer [88]
miR-34a/b/c ↓	(+) tumor initiation in mouse *Kras; Trp53* transgenic lung cancer [51] (+) tumor initiation by repressing inhibin subunit beta B and AXL in mouse *Apc* transgenic colorectal cancer [89]	(+) chemoresistance to fludarabine through p53 inactivation and apoptosis resistance in human chronic lymphocytic leukemia [90]

EGFR: epidermal growth factor receptor; Akt: Akt Serine/Threonine Kinase; MAPK: mitogen-activated protein kinase; MEK: Mitogen-activated protein kinase kinase; ERK: extracellular-signal-regulated kinase; PI3K: phosphatidylinositol 3-kinase; AXL: AXL receptor tyrosine kinase; Apc: adenomatous polyposis coli; VHL: Von Hippel–Lindau; mTOR: mammalian target of rapamycin; ↑: upregulation; ↓: downregulation; (+): promotion; (−): repression; N.A.: not available.
